# 1992. Two Decades of Lyme Disease in the U.S. Veterans Health Administration Detected Via Automated Surveillance Methodology, October 1999 - September 2022

**DOI:** 10.1093/ofid/ofad500.119

**Published:** 2023-11-27

**Authors:** Gina Oda, Cynthia Lucero-Obusan, Patricia Schirmer, Mark Holodniy

**Affiliations:** Department of Veterans Affairs, Palo Alto, CA; Department of Veterans Affairs, Palo Alto, CA; Department of Veterans Affairs, Palo Alto, CA; Department of Veterans Affairs, Palo Alto, CA

## Abstract

**Background:**

Lyme disease is the most common vector-borne disease in the U.S. Incidence of Lyme disease is rising, partly due to climate change, and is likely underreported. We evaluated an automated method to analyze Lyme disease trends over two decades in the Veterans Health Administration (VHA).

**Methods:**

We included individuals receiving VHA outpatient and/or inpatient care during fiscal years (FY) 2000-2022 (10/1/1999 – 9/30/2022). Automated case-finding was performed via ICD 9/10 Lyme Disease code plus a prescription for ≥7 days of appropriate antimicrobial treatment within 30 days of encounter AND/OR confirmatory lab testing (Table 1). Data was extracted from VHA Corporate Data Warehouse. We calculated FY incidence and prevalence per 10,000 individuals in VHA care and analyzed demographic trends. Positive Predictive Value (PPV) of case-finding methodology and clinical characteristics of identified cases was determined by review of 75 randomly selected case records.

**Table 1.**
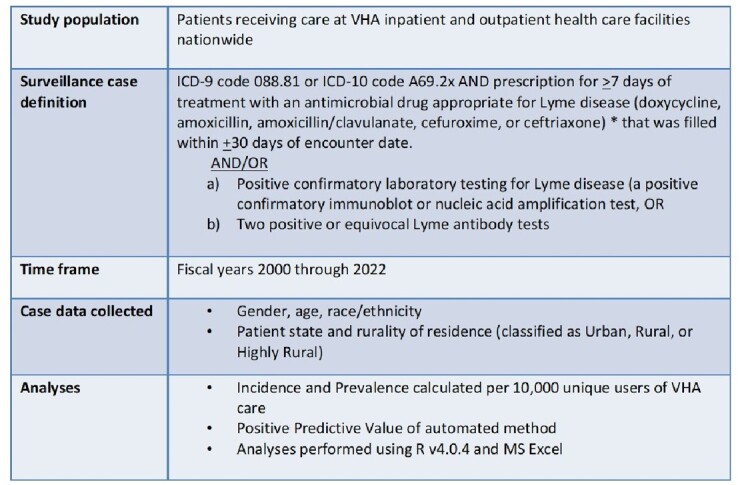
Methodology

*Nelson, C.A., et al. Emerging Infectious Diseases. Vol. 21, No. 9, September 2015

**Results:**

Automated methodology detected a total of 24,349 Lyme disease cases. Incidence rose steadily, peaking at 3.25 per 10,000 in FY 2017, with prevalence reaching 32.72/10,000 in FY 2022 (Fig. 1). States with highest average incidence per 10,000 in care were RI (12.65), ME (10.61), VT (9.81), and WI (8.51). Distribution of Lyme disease incidence expanded nationally, particularly in the Northeast and Northern Midwest. Demographic groups with highest average incidence per 10,000 in care included individuals in rural/highly rural areas (2.41), non-Hispanic Whites (2.26), age groups 55-64, 65-74, and 25-34 (2.30, 2.20, and 2.08, respectively), and males (1.95) (Fig. 2). Overall PPV of automated case-finding was 85.3%, with ICD 9/10 code + appropriate antimicrobial treatment AND confirmatory lab testing being the most accurate method (PPV 100%) (Table 2).

**Figure 1.**
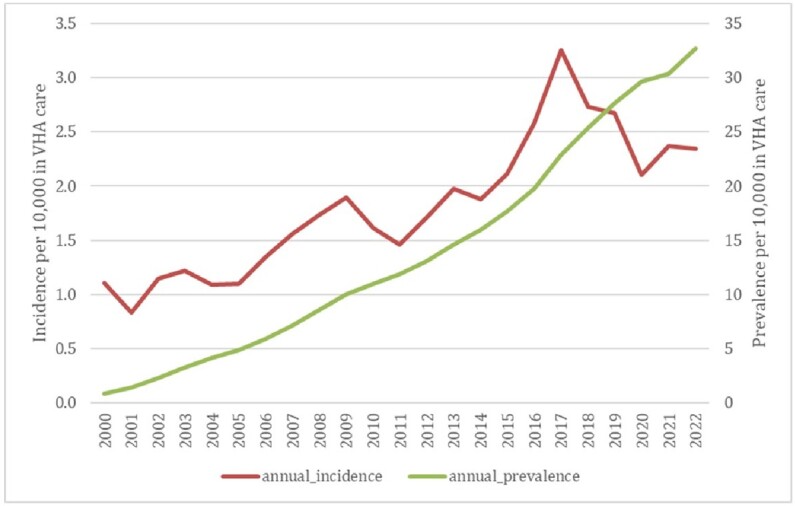
Incidence and Prevalence of Lyme Disease in VHA, Fiscal Years 2000 - 2022

**Figure 2.**
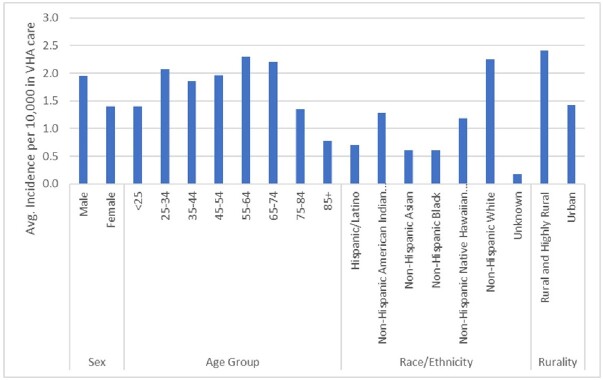
VHA Lyme Disease Average Incidence rates by Demographic Variable, FY 2000 - 2022

**Table 2.**
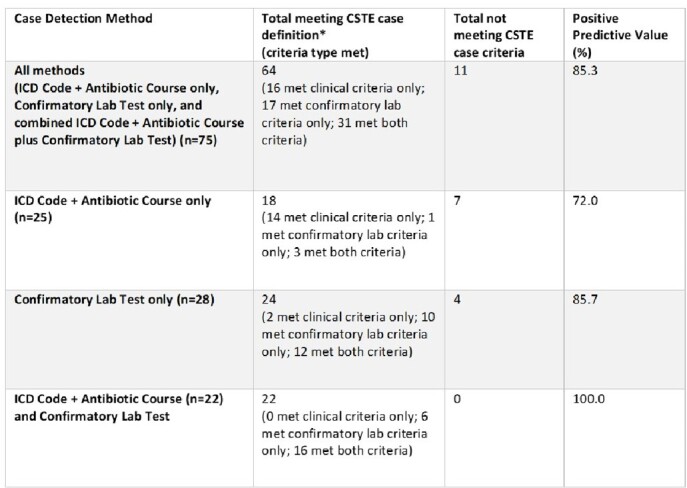
Positive Predictive Value of Automated Case-Finding, N=75

*CSTE case definition = the current surveillance definition used by the Centers for Disease Control and Prevention Lyme Disease (Borrelia burgdorferi) 2022 Case Definition | CDC, based on 2021 Council of State and Territorial Epidemiologists Position Statement 21-ID-05: “Modification of Lyme Disease Case Definition”.

**Conclusion:**

Automated surveillance methodology for Lyme disease provided an efficient and reasonably accurate estimation of the epidemiology of Lyme disease in VHA. The VHA pattern of Lyme disease geographic expansion in recent years is consistent with CDC national reporting. Providers should be aware of Lyme disease as an emerging threat.

**Disclosures:**

**All Authors**: No reported disclosures

